# Influence of extracorporeal shock wave therapy (ESWT) on bone turnover markers in organisms with normal and low bone mineral density during fracture healing: a randomized clinical trial

**DOI:** 10.3205/iprs000119

**Published:** 2017-12-18

**Authors:** Christoph Wölfl, Laura Schuster, Bernd Höner, Sarah Englert, Roman Klein, Christoph Hirche, Matthias Münzberg, Paul Alfred Grützner, Ulrich Kneser, Leila Harhaus

**Affiliations:** 1 Department of Orthopedic and Trauma Surgery, Marienhaus Klinikum Hetzelstift, Neustadt/Weinstrasse, Germany; 2 Departement of Surgery, Evangelisches Krankenhaus Karlsruhe Rüpprurr, Karlsruhe, Germany; 3 SRH University Heidelberg, Department of Social Sciences and Law, Heidelberg, Germany; 4 Department of Plastic-, Reconstructive and Handsurgery, Burn Care Centre, Department of Plastic Surgery of Heidelberg University, BG Traumacenter Ludwigshafen, Ludwigshafen, Germany; 5 Department of Orthopedic and Trauma Surgery, BG Traumacenter Ludwigshafen, Ludwigshafen, Germany

**Keywords:** ESWT, extracorporeal shock wave therapy, bone turnover markers, fracture healing, osteoporosis, bone mineral density, vitamin D3, parathyroid hormone, TRAP5b, BAP, beta-CTX

## Abstract

**Background:** Low bone mineral density (BMD) leads to metaphyseal fractures, which are considered of delayed, qualitatively reduced healing resulting in prolonged care phases and increased socioeconomic costs. Extracorporeal shockwave therapy (ESWT) is already approved to support bone healing of pseudarthrosis and delayed unions. With this study, we examined its influence on bone turnover markers (BTM) during fracture healing in patients with low and normal BMD.

**Methods:** Within a period of 2 years, patients with a metaphyseal fracture of the distal radius or the proximal humerus, requiring surgical osteosynthesis were included into the study. Patients were randomized within their fracture groups whether they received ESWT after surgery or not. ESWT was applied once after surgery with an energy flux density (EFD) of 0.55 mJ/mm² à 3000 shockwaves. In addition, serum levels of vitamin D3, parathyroid hormone (iPTH), bone alkaline phosphatase (BAP), c-telopeptide of type-I-collagen (β-CTX) and serum band 5 tartrate-resistant acid phosphate (TRAP5b) were determined before surgery and post-operatively in week 1, 4, 8, 52. T-score levels as an indicator of the BMD were measured with dual-energy X-ray absorptiometry (DXA).

**Results:** 49 patients (40 females, 9 males; mean age 62 years) with fractures of the metaphyseal distal radius (n=25) or the proximal humerus (n=24) were included in the study. The follow-up time was one year. 24 of them were diagnosed of having low BMD, whereas 25 had a normal BMD. During follow-up time serum levels of bone turnover markers, as well as vitamin D3 and iPTH, showed no significant changes; however, ESWT approaches the decreased serum levels of patients with low BMD to the level of healthy organisms.

**Conclusions:** ESWT as treatment option of fractures in patients with low BMD can lead to an equilibration of levels of bone turnover markers to the levels of patients with normal BMD.

## Zusammenfassung

Die Osteoporose und die damit verbundenen Folgen nehmen eine immer bedeutendere Rolle ein. Durch reduzierte Knochendichte bedingte Frakturen heilen langsamer und qualitativ minderwertiger. Aus der Pseudarthrosenbehandlung ist bekannt, dass die Anwendung der Extrakorporalen Stoßwellentherapie EWST einen positiven Effekt auf die Knochenbruchheilung hat.

Der Einfluss der Stimulation durch ESWT auf die Frakturheilung bei normaler und erniedrigter Knochendichte wird in dieser Studie untersucht.

Zwischen 2011 und 2013 wurden insgesamt 49 (40 w, 9 m) Patienten (>45, <70 Jahren) mit frischer, operationspflichtiger metaphysärer Fraktur des distalen Radius oder des proximalen Humerus nach Stolpersturz in die Studie eingeschlossen. Jeweils 12 von ihnen (Studiengruppe) erhielten unmittelbar nach der Osteosynthese standardisiert ESWT (0.55 mJ/mm², 3000 Stoßwellen). Die Zuteilung wurde verblindet randomisiert.

Vor Einschluss in die Studie nach Leitlinie, wurde eine umfassende Anamnese erhoben. Zusätzlich fand die Einschätzung der Risikofaktoren für Osteoporose mittels LOS-Bogen (Ludwigshafener Osteoporose Screening Bogen) statt. Lag bereits eine behandelte Osteoporose vor, so galt dies u.a. als Ausschlusskriterium zur Studienteilnahme.

Bei jedem Patienten erfolgten Blutentnahmen präoperativ sowie postoperativ in Woche 1, 4, 8 und 52 mit Auswertung folgender Parameter: Vitamin D3, Parathormon intakt, BAP, TRAP5b und CTX. Mittels dual-energy X-ray-absorptiometry (DXA) wurde die Knochendichte (T-Wert) in Woche 1 und 52 bestimmt, zudem erhielten die Patienten einmalig ein Röntgen der Lendenwirbelsäule zum Ausschluss von Sinterungsfrakturen. Hierdurch ließ sich gegebenenfalls die Erstdiagnose „Osteoporose“ stellen. Verglichen wurde der Verlauf der Knochenstoffwechselparameter mit und ohne ESWT-Stimulation zwischen den Patienten mit normaler und erniedrigter Knochendichte.

Im Verlauf von Vitamin D3 und Parathormon zeigten sich keine signifikanten Unterschiede. Bei BAP konnte kein Einfluss auf die Knochendichte sowie einen Einfluss durch die ESWT aufgezeigt werden.

Hingegen konnte bei den Verläufen von TRAP5b nachgewiesen werden, dass die Werte unabhängig der Behandlungsgruppe und Knochendichte signifikant absanken (p<0,001). Des Weiteren hatten die Patienten mit erniedrigter Knochendichte signifikant höhere Werte (p=0,007). Es zeigte sich eine signifikante zweiseitige Interaktion zwischen Behandlungsgruppe*Zeit (p=0,001), in der Kontrollgruppe blieben die Werte konstant, in der Stoßwellengruppe stiegen sie an. Bei den Verläufen von CTX kam es ebenfalls zu einem signifikanten Abfall über die Zeit in allen Gruppen (p=0,006). Des Weiteren konnte ein Trend für einen zweiseitigen Interaktionseffekt der Behandlungsgruppe*Zeit (p=0,080) nachgewiesen werden, die ESWT-Gruppe blieb mit den Werten stabil, in der Kontrollgruppe gab es einen Abfall.

Auch wenn die hier erzielten Ergebnisse keinen signifikanten Einfluss der ESWT auf die Knochenheilung widerspiegeln, so zeigte sich doch eine tendenzielle Aktivierung der osteokatabolen Stoffwechselfaktoren. In tierexperimentellen Studien konnte nachgewiesen werden, dass der positive Einfluss auf die Frakturheilung mit der Anzahl der Wiederholungen der ESWT-Applikation steigt. In Folgestudien soll daher eine optimierte Anwendung der ESWT ermittelt werden.

## Background

Osteopenia, Osteoporosis, osteoporotic fractures and the associated healthcare costs are going to be one of the main health problems in our aging society [1], [2], [3]. Fractures that occur in bones with low bone mineral density (BMD) are considered of delayed healing and a difficult management of stabilization [2]. To prevent complications after these fractures, to improve fracture healing, and to reduce mortality, morbidity and healthcare costs, supporting therapies in addition to standard osteosynthesis need to be established. One such therapy might be extracorporeal shock wave therapy (ESWT).

By now, ESWT is commonly used in orthopedics for treatment of plantar fasciitis, calcific tendinitis of the shoulder, delayed union or non-union of long bones among others [4], [5], [6], [7]. 

Shock waves are acoustical pulses generated outside of the body (extracorporeal), applied on the skin, spread through the different kind of tissues and take effect at defined places inside the body [8], [9]. In various studies, the influence of ESWT on bone metabolism was examined but the exact pathway of these specific effects still remains subject of further examination. 

An increased cortical volume and higher trabecular connectivity was observed after stimulation with ESWT and that again may lead to improved biomechanics of the bone [10]. On molecular level a significantly rising amount of Fibroblast-Growth-Factor-2 (FGF-2) has been found after fibroblasts and osteoblasts received ESWT [11].

The time course of bone turnover markers, such as bone alkaline phosphatase (BAP), transforming growth factor β1 (TGF-β1), c-telopeptide of type-I-collagen (β-CTX), serum band 5 tartrate-resistant acid phosphate (TRAP5b), during fracture healing is a diagnostic method, which allows insight into ongoing processes and gives the possibility to recognize early an impaired healing course. A change of time courses of these turnover markers during fracture healing of bones with low BMD has already been observed [12], [13], [14]. In the present study, we want to evaluate whether external stimulation with ESWT has an impact on the healing process in bone with normal and low BMD. We therefore examined the time course of bone turnover markers to detect the influence of ESWT on the particular bone metabolism during fracture healing processes. 

## Methods

The study was approved by the local ethical committee (Mainz, Germany) (837.368.10 (7377)). The study was conducted according to the principles of the declaration of Helsinki. All data were analyzed anonymously with cypher. All patients gave their written informed consent.

Between March 2011 and March 2013 all patients with a metaphyseal fracture of the distal radius (DR#) or the proximal humerus (PH#) that required a surgical osteosynthesis were asked to participate in the study. 

Exclusion criteria were pharmaceutical treatment of osteoporosis, conservatively treated fractures, pathological fractures except osteoporotic fractures, malignancy or systemic diseases with skeletal involvement, immobilization/confinement to bed, prosthetic treatment in the course and non-compliance of the patient. Patients received full information about the study. Patient’s medical history was documented and extended with the LOS-Questionnaire (“Ludwigshafen Osteoporosis Screening” – Questionnaire) [15]. 

In each group (DR# and PH#) half of the included patients received a standardized ESWT immediately after osteosynthesis (state of the art). The allocation to study- or control-group was randomized. ESWT was applied during anesthesia once with an energy flux density (EFD) of 0.55 mJ/mm² à 3000 shock waves on the fracture area after suture from dorsal side (DR#) and ventral side (PH#), respectively [16], [17], [18]. We used the device “Duolith” with F-SW handpiece of STORZ MEDICAL AG (Tägerwilen, Switzerland). 

The control-group received no further treatment except osteosynthesis. 

To measure the bone mineral density (BMD) we used dual-energy X-ray absorptiometry (DXA; Lunar iDPX, GE Medical Systems Germany, Solingen, Germany) based on Encore TM Version II.X software. Within one week after surgery and again after one year, all patients were examined by a standardized protocol measuring the density of the lumbar spine and both femoral necks. The unit of measurement describes the t-score, which is the standard deviation of the mean value of the peak bone mass in young people. A t-score between –1 and –2.5 is classified as osteopenia and a t-score smaller than –2.5 as osteoporosis by the World Health Organization (WHO) [3].

In addition, an X-ray of the lumbar spine was performed in anterior/posterior and lateral view to exclude any alterations of the vertebral bodies in the first week after surgery.

Blood samples (EDTA, serum) were taken preoperatively as well as postoperatively at week 1, 4, 8 and 52 after surgery and ESWT or surgery alone with the patient in a fasting state. Therewith, the time-course of vitamin D3, intact parathyroid hormone (iPTH), bone formation marker bone alkaline phosphatase (BAP) as well as osteocatabolic markers TRAP5b and β-CTX were monitored. 

Quantitative measurements were obtained by using IDS-iSYS Ostase^®^BAP Assay (ISYS, IDS) for BAP, BoneTRAP^®^ Assay (DS2, DYNEX) for TRAP5b, both are ELISA. The measurements of vitamin D3, iPTH and β-CTX were run by a fully automatic machine using the measuring system E170 Modular of Roche Diagnostics (Germany).

A radiological follow-up could have been performed by approximately 50% of all patients four weeks postoperatively (+/– 3 days). Exemplarily two patients of them with ESWT were matched to two patients without ESWT regarding fracture type and t-score. 

The study was designed prospectively as a randomized clinical trial. Due to the results of the DXA measurements all patients were divided into a normal BMD group and a low BMD group by using a t-score of –2 as a cut-off value. This cut-off value was used to detect not only patients with manifest osteoporosis, but already the patients with low BMD. We compared each time-course of the blood values on the one hand, patients with low and normal BMD who received ESWT (study group) and on the other hand, patients with low and normal BMD without receiving ESWT (control group).

ANOVA (4-factor analysis) and Tukey Kramer-post hoc test were performed using the software SPSS 20.0.0 (IBM Germany, Munich), results were exported to Microsoft Excel for presentation. At each point we used the mean values of the blood levels.

A value of p≤0.05 was considered to be significant, p≤0.01 very significant and p≤0.001 highly significant.

Table 1 (Tab. 1) gives an overview on the study design.

## Results

Between March 2011 and March 2013 49 patients (40 females, 9 males) with a mean age of 62 years (range: 46 and 76) with fractures of the metaphyseal distal radius or the proximal humerus have been included in the study. The follow-up time was one year. 25 of them suffered from a fracture of the radius and 24 from a fracture of the humerus. By using DXA measurement, the patients were diagnosed of having normal or low BMD. Hence, in normal BMD group n was = 25 (ESWT = 14, control-group = 11), whereas in low BMD group n was = 24 (ESWT = 10, control-group = 14). The distribution of fractures, age, gender and t-score are illustrated in Table 2 (Tab. 2).

Thus, the comparison is shown between study group and control group and the particular effects of the time courses of the blood values in patients with low or normal BMD.

The results of the laboratory analyses are presented in Figure 1 (Fig. 1), Figure 2 (Fig. 2), Figure 3 (Fig. 3), Figure 4 (Fig. 4), Figure 5 (Fig. 5), Figure 6 (Fig. 6), Figure 7 (Fig. 7), Figure 8 (Fig. 8), Figure 9 (Fig. 9), Figure 10 (Fig. 10). 

The time courses of vitamin D3 in the control group both increased steadily, whereas the last value of the patients with normal BMD decreased below the value of the patients with low BMD. In ESWT group the courses nearly remained stable except the last value of patients with low BMD, which increased. Apparently, all values of vitamin D3 ranged at the lower levels of reference. 

In iPTH control group the time courses of patients with low and normal BMD showed a similar course: from preoperatively to week 1 it decreased, after that it slightly increased. The time course of patients with low BMD always lay over the one of patients with normal BMD. Likewise, similar time courses were detected in the ESWT group although from week 8 postoperatively low and normal BMD courses show nearly the same values. Overall, the time courses of vitamin D3 and iPTH showed no significant differences in the groups with and without ESWT in normal and low BMD. An influence of ESWT on vitamin D3 and parathyroid hormone could not be observed.

In the control group, the time courses of BAP showed no significant difference and a similar course. Initially, BAP decreased slightly. In week 4 after surgery, there was an increase to the highest level of detection. Afterwards, the course nearly remained stable in patients with normal BMD. Whereas BAP decreases again in patients with low BMD. All values remained within the reference level. In the study group, both BMD groups showed an increase between the dates of the first blood withdrawal preoperatively until week 4 postoperatively. Furthermore, in patients with normal BMD in week 4 postoperatively a peak was shown (not significantly) compared to patients with low BMD. Afterwards, both parameters decreased to the level of measurement 1. All values remained within the reference range, as well. 

Regarding the time courses of TRAP5b in the control group, again, there was no significance seen between patients with low or normal BMD. Even though a significant difference could not be proved in the study group, the time courses of both BMD levels increases at the beginning until week 4, which distinguishes from the time courses in the control group. Thereafter, both parameters decreased below the starting level. Both control group and study group, the course of patients with low BMD lay above the course of patients with normal BMD. In all groups the values remain within the reference level. 

In the control group, the time course of β-CTX decreased in both BMD levels at the beginning. After that, the course of patients with low BMD showed no detectable action. However, the course of β-CTX in patients with normal BMD increases in week 4 again and then decreases without significance. 

In the study group, a decrease in the first week postoperatively does not occur, instead both time courses nearly remained stable and levels decreased below the starting point level. The time course of patients with low BMD exceeds the time course of patients with normal BMD at all points of measuring. Again, all values remained within the reference range. 

X-ray follow-up could be performed in approximately 50% of cases at 4 weeks +/– 3 days and at one year after surgery. After one year, all fractures were consolidated completely. In one case, a complication could be observed with sintering of the proximal humerus, which was treated conservatively. To detect the early effects of ESWT on fracture healing, special regard was taken onto the X-rays at 4 weeks after surgery and patients were matched as described above. Table 3 (Tab. 3) shows two exemplarily matched cases that highlight a faster healing process of fractures with ESWT compared to fractures without ESWT in low and normal BMD levels. 

## Discussion

Due to an increasingly aging society, more and more people suffer from low bone mineral density and its final stage osteoporosis. Low bone mineral density leads to different fractures, mostly in the distal radius, proximal humerus, femur or vertebrae and these are associated with increasing socio-economic health costs [15], [19], [20], [21], [22], [23]. In postmenopausal women at the age of 50–60, the prevalence of osteoporosis is at about 15%. At the age of over 70 years, the prevalence rises up to 45%. In men at the age of 50 to 60 years, the prevalence of a low BMD is at 2.4% and increases to 17% at the age over 70 [24], [25], [26]. Since the healing of fractures occurring in bone with low BMD is associated with poorer results, re-fractures and delayed healing, it is immensely important to improve fracture healing in bones with low BMD [2], [27], [28]. Therefore, it is necessary to provide insight in the ongoing bio-molecular healing processes in bones with low BMD in comparison to bones with normal BMD [29], [30], [31], [32]. 

The use of extracorporeal shockwave therapy has been established many years ago: On the one hand, it is applied in urological diseases such as kidney stones, on the other hand, it is successfully used in the treatment of plantar fasciitis, calcific tendinitis of the shoulder, delayed union or non-union of long bones [33], [34], [35]. Elster et al. described a successful treatment with ESWT of tibia non-unions and suggest that ESWT affects the development of tissues and bone repair [6]. 

In previous studies we examined the time course of those parameters during fracture healing in patients with normal or low bone mineral density in detail. We found partly significantly different time courses of up-regulation of specific alkaline phosphatase (BAP) and transforming growth factor β1 (TGF-β1), as well as the bone resorption markers crosslinked C-telopeptide of type-I-collagen (β-CTX) and serum band 5 tartrate-resistant acid phosphate (TRAP5b). In addition, the time courses of vitamin D3 and parathyroid hormone (iPTH) have been examined because both parameters are important modulators of calcium and bone homeostasis, but no significant differences could be seen between the groups of normal and low BMD [12], [13]. Vitamin D3 and iPTH are affected of other metabolic pathways, thus it is not surprising that an influence of ESWT could not be shown here. 

BAP is a product of osteoblasts and does reflect their activity. It is a marker for bone formation. In this study, there is a steady increase in both BMD levels in the ESWT group until in week 4. Although no significance is shown, our results suggest that osteoblasts in patients with normal BMD seem to be more activated by ESWT than in low BMD. 

Especially osteoclasts and macrophages produce TRAP5b, an osteocatabolic turn-over marker. TRAP5b correlates with the amount of osteoclasts. If there is an increased bone resorption, the amount of osteoclasts increased as well. Hence, TRAP5b rises with higher bone resorption [36]. TRAP5b is elevated in patients with osteoporosis, which is reflected in both groups in this study. In the control group, no difference in the time course is seen in patients with low BMD, whereas in the study group TRAP5b is more activated than in patients with normal BMD. Furthermore, it reaches the level of patients with normal BMD at the latest measurement in this study. This might indicate an improved bone homeostasis of the organism with low BMD through ESWT.

The osteocatabolic marker β-CTX is used to evaluate the activity of bone resorption and to monitor an antiresorptive therapy. The organic matrix of bone mostly consists of collagen type I that is split into its N- and C-terminal telopeptides (CTX) during bone resorption by osteoclasts. The β-CTX is released in the bloodstream. Elevated concentrations are found in patients with increased bone resorption [37]. A significant change of β-CTX by ESWT could not be observed. 

The radiologic examination is the most important assessment to clinically evaluate the healing process of fractures. Due to the special structure of the German health system, the complete radiological follow up of outpatient cases was not possible in our hospital. Thus, we only could perform X-rays in half of the cases. In the exemplarily matched pairs, we could find at the time point of four weeks after surgery that the healing process was visibly accelerated in patients who received ESWT compared to patients who did not receive ESWT. However, a final statement whether ESWT can improve and accelerate fracture healing requires further studies.

## Limitations

This study was designed and planned carefully, yet it bears some limitations. First, X-ray controls could not be performed of all patients due the structure of the German outpatient health system. Patients who developed complications or needed revision surgery should have been treated at our hospital in any case, since we are the largest trauma center in the region. We may therewith assume that patients without X-ray controls did not suffer from procedure-related complication. 

Second, with 49 patients the size of the groups is relatively small and inhomogeneous concerning age, gender and BMD. On the other hand, this patient structure represents the typical population of patients suffering metaphyseal fractures. 

## Conclusion

Extracorporeal shock wave therapy as treatment option of fractures in patients with low BMD can lead to an approximation of levels of bone turnover markers to the level of patients with normal BMD and therewith may help to improve and accelerate fracture healing in low BMD organisms. 

## Abbreviations

- BMD = Bone mineral density
- BTM = Bone turnover markers
- DXA = Dual X-ray absorptiometry
- ESWT = Extracorporeal shock wave therapy
- DR# = Distal radius fracture
- PH# = Proximal humerus fracture
- LOS-Questionnaire = “Ludwigshafen Osteoporosis Screening” – Questionnaire
- iPTH = Intact parathyroid hormone
- ß-CTX = C-telopeptide of type-I-collagen
- TRAP5b = Serum band 5 tartrate-resistant acid phosphate
- BAP = Bone alkaline phosphatase
- EFD = Energy flux density

## Notes

### Competing interests

The authors declare that they have no financial or non-financial competing interests.

### Ethics approval and consent to participate

The study was approved by the local ethical committee (Mainz, Germany) (837.368.10 (7377)). The study was conducted according to the principles of the declaration of Helsinki. All data were analyzed anonymously with cypher. All patients gave their written informed consent.

### Authorship

CW and LS share the first authorship.

CW substantially developed the design of the study, performed the surgeries and the ESWT applications. LS performed the collection and statistical analysis of the data and wrote the main part of the manuscript. BH performed substantial parts of the statistical analysis and data interpretation. SE performed the inclusion and preparation of the patients and patient consent forms. CN participated in the surgeries and follow up examinations. CH substantially assisted in interpretation of the data and writing of the manuscript. MM took care for the patient management, the exact determination of blood sample collections and data interpretation. PAG and UK conceived of the study and participated in its design and coordination and helped to draft the manuscript. LH coordinated the study, designed the manuscript and wrote substantial parts of it. All authors read and approved the final manuscript.

### Acknowledgements

We thank all colleges who participated in the surgeries and care of the patients. Thanks are due to Gina Mackert (M.D.) as native speaker for her language editing. 

## Figures and Tables

**Table 1:**
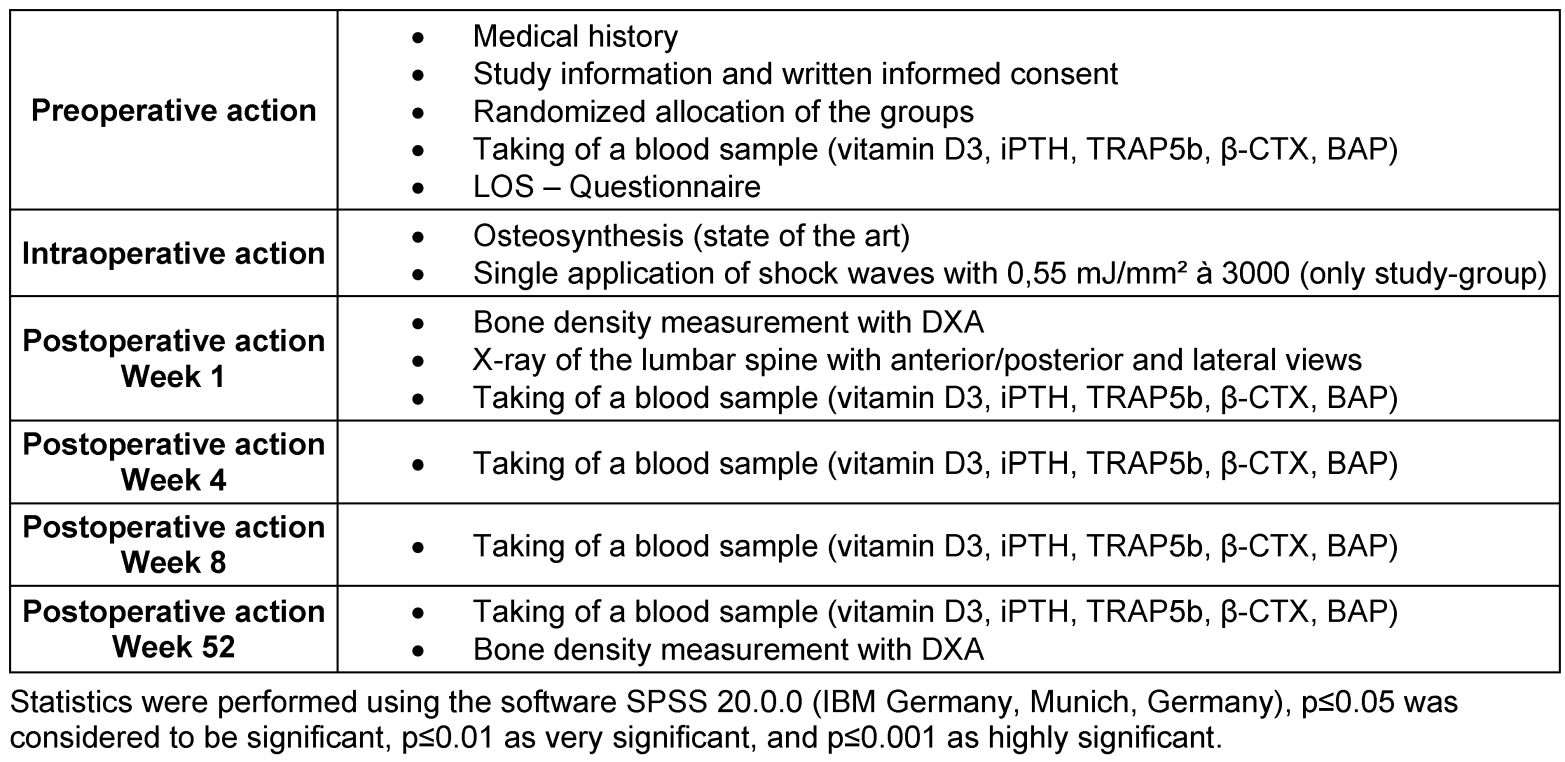
Study protocol

**Table 2:**
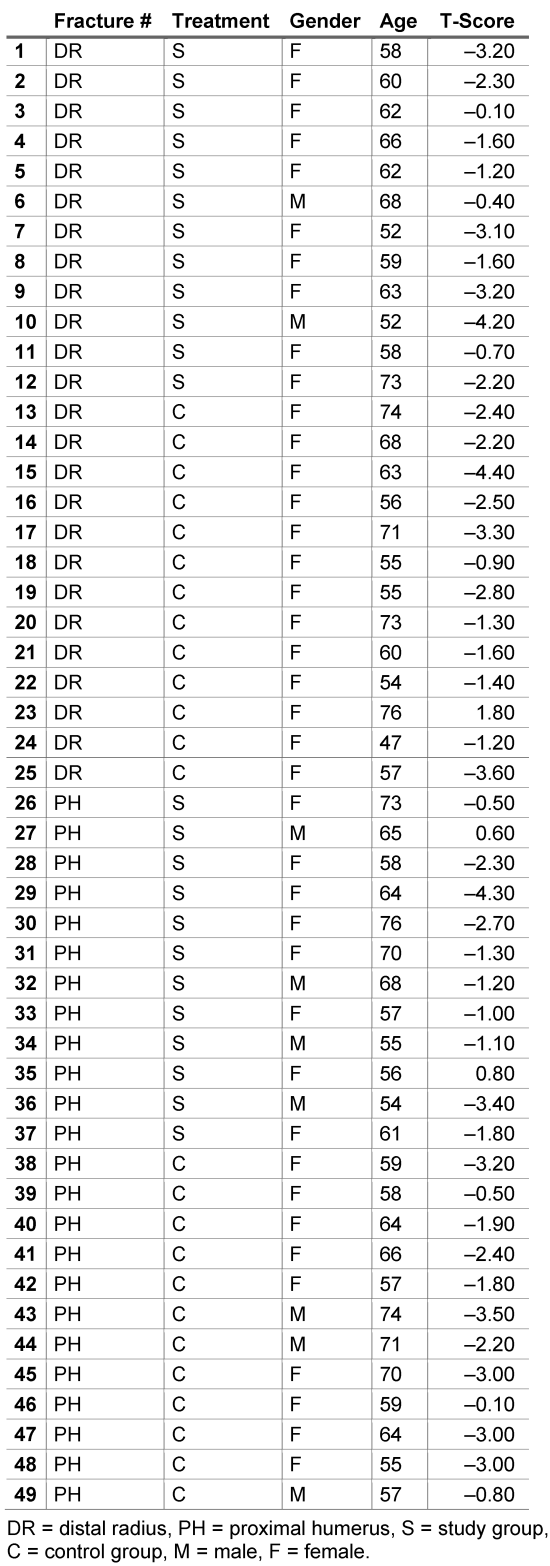
Demographic data

**Table 3:**
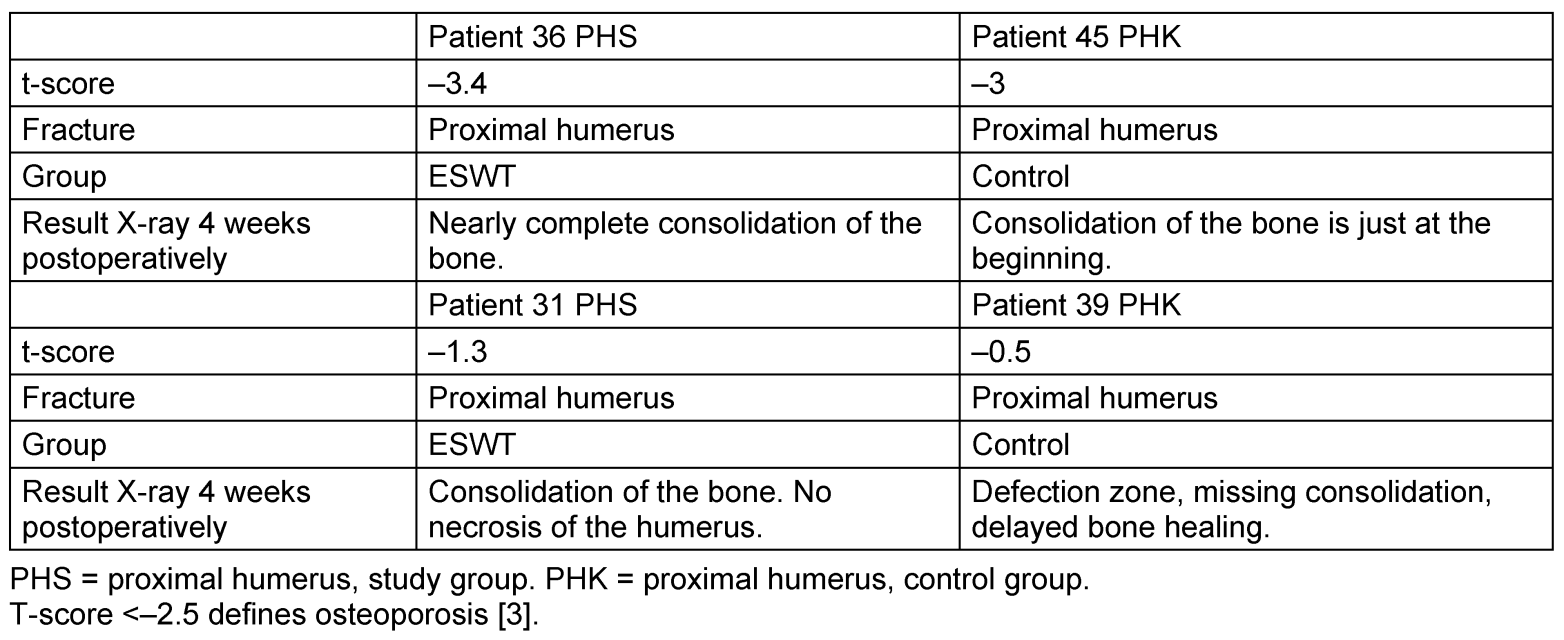
Table with two exemplarily matched pairs of patients and their X-rays 4 weeks after surgery

**Figure 1:**
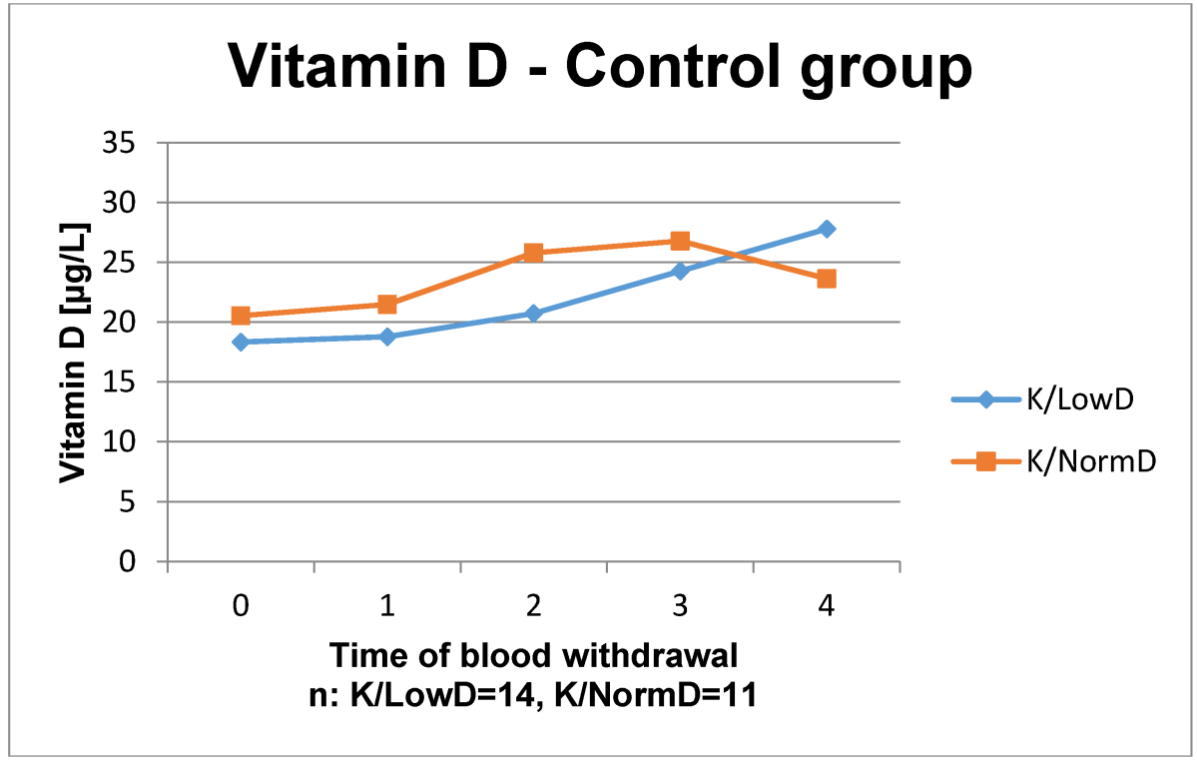
Time course of vitamin D in patients without ESWT

**Figure 2:**
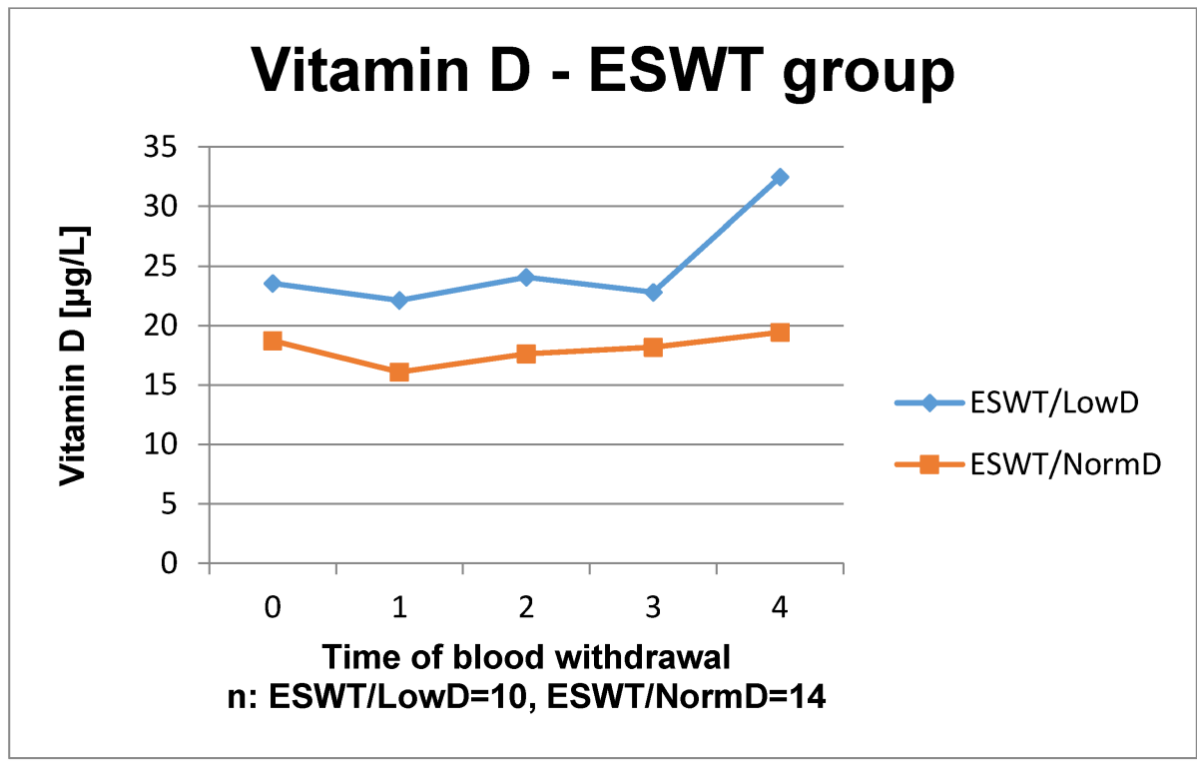
Time course of vitamin D in patients with ESWT Reference range: 20–70 µg/l

**Figure 3:**
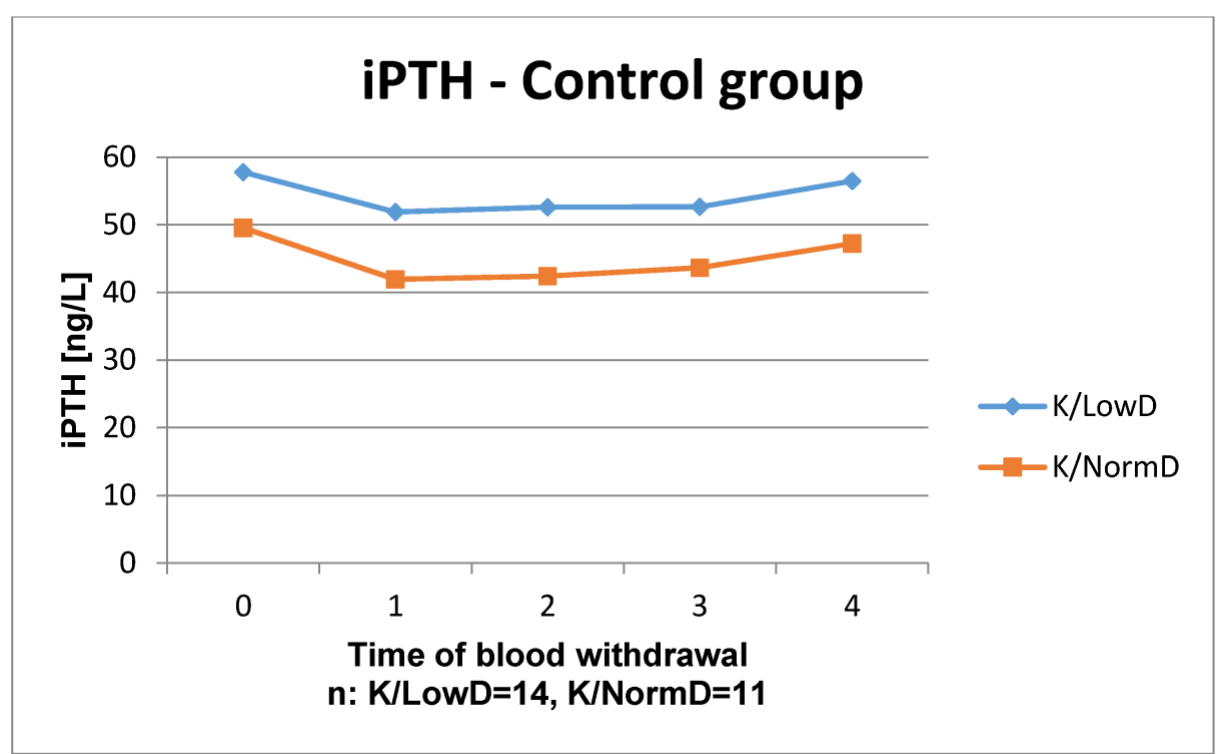
Time course of iPTH in patients without ESWT

**Figure 4:**
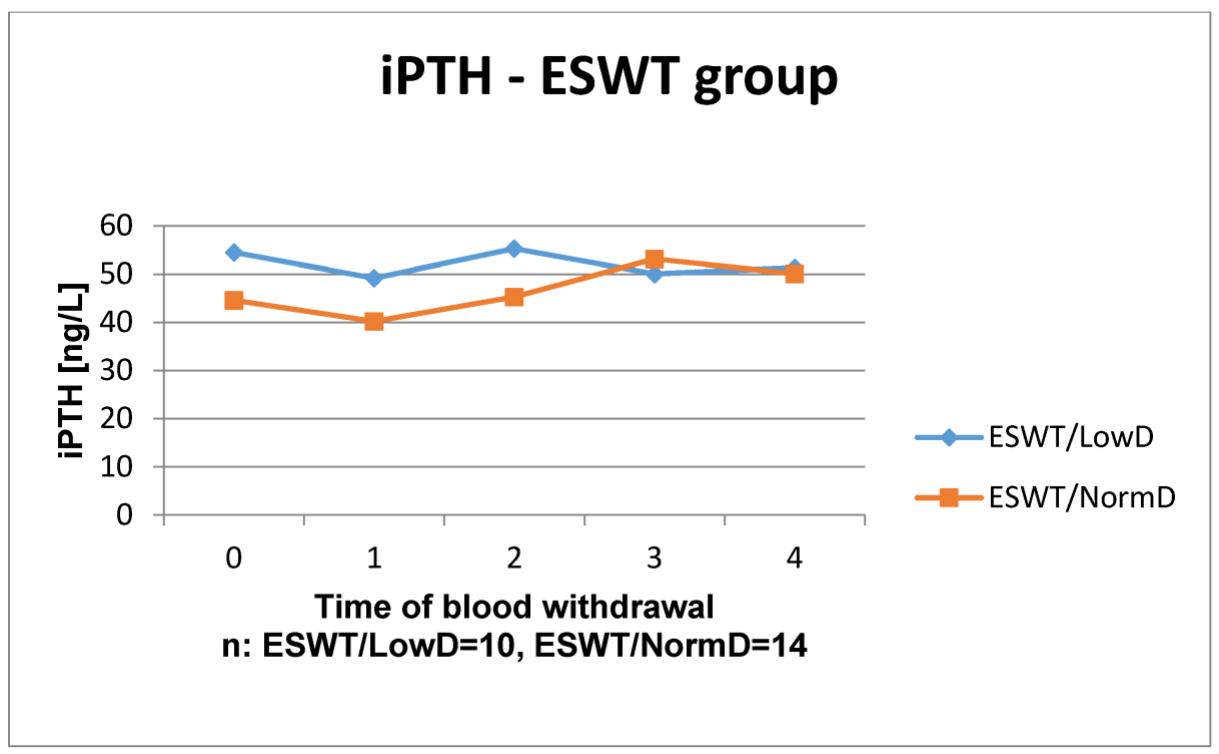
Time course of iPTH in patients with ESWT Reference range: 11–43 ng/l

**Figure 5:**
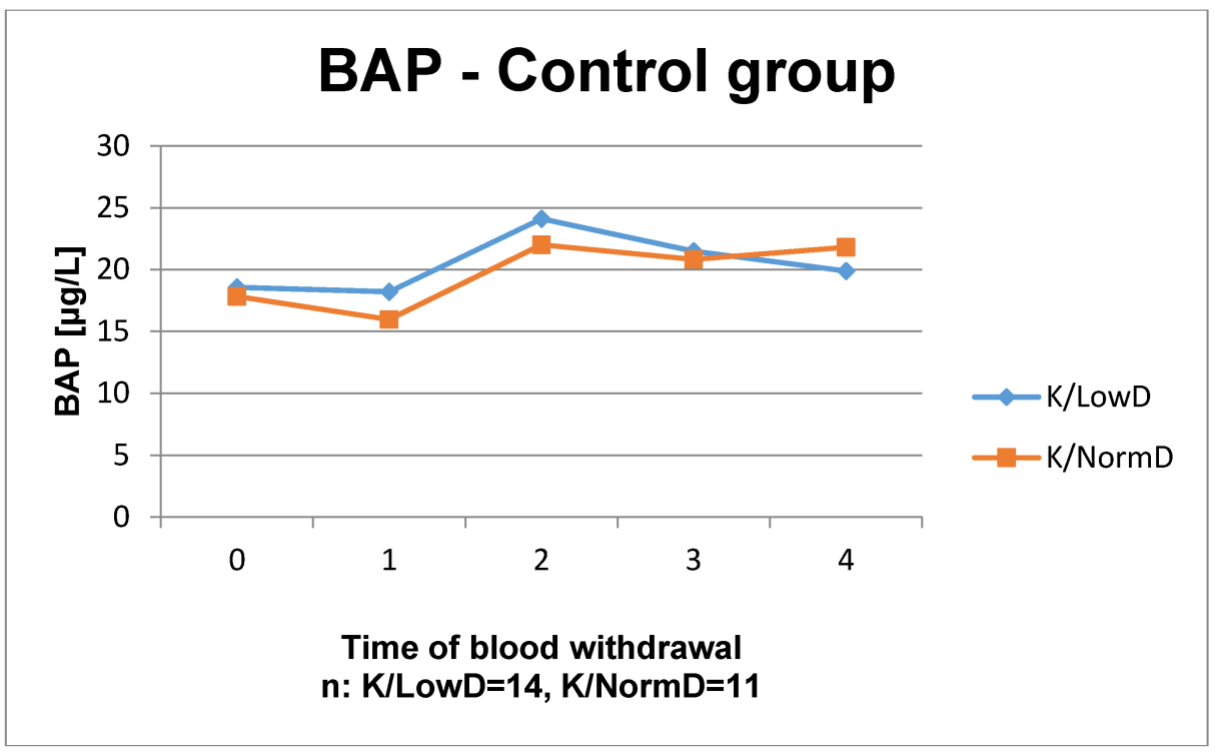
Time course of BAP in patients without ESWT

**Figure 6:**
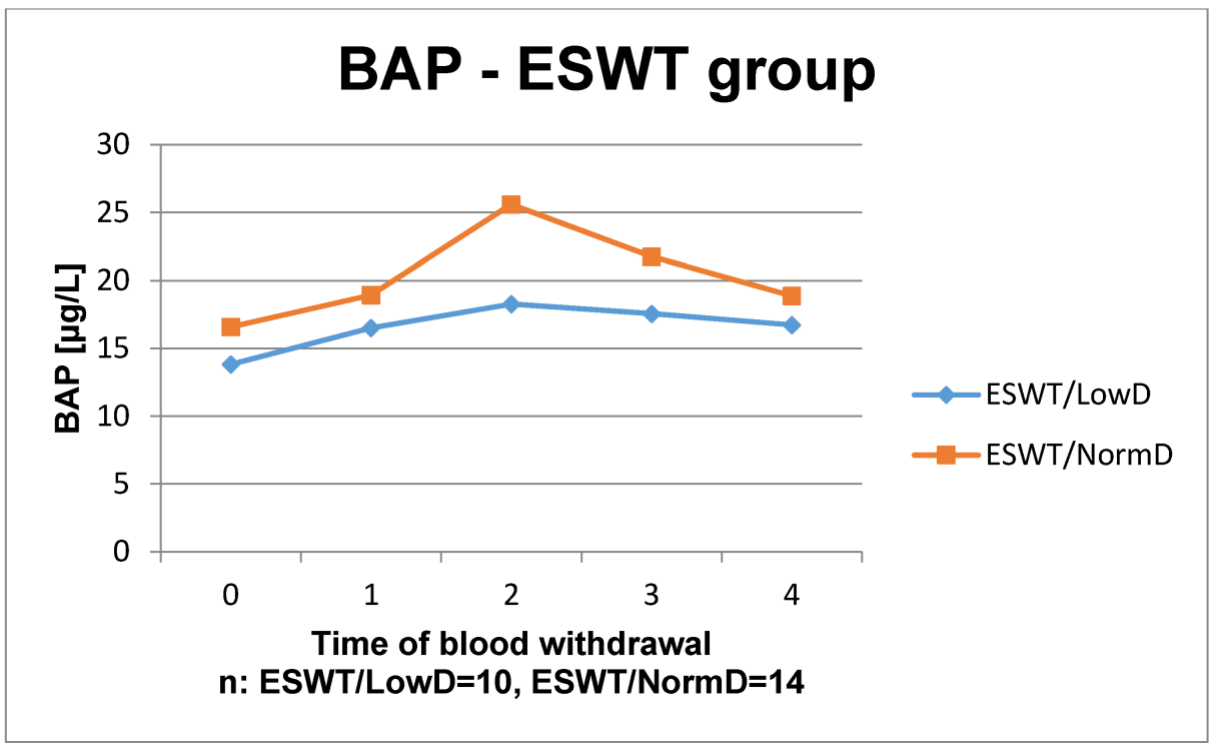
Time course of BAP in patients with ESWT Reference range: female (F): 6–22.7 µg/l (premenopausal), male (M): 7.5–26.1 µg/l

**Figure 7:**
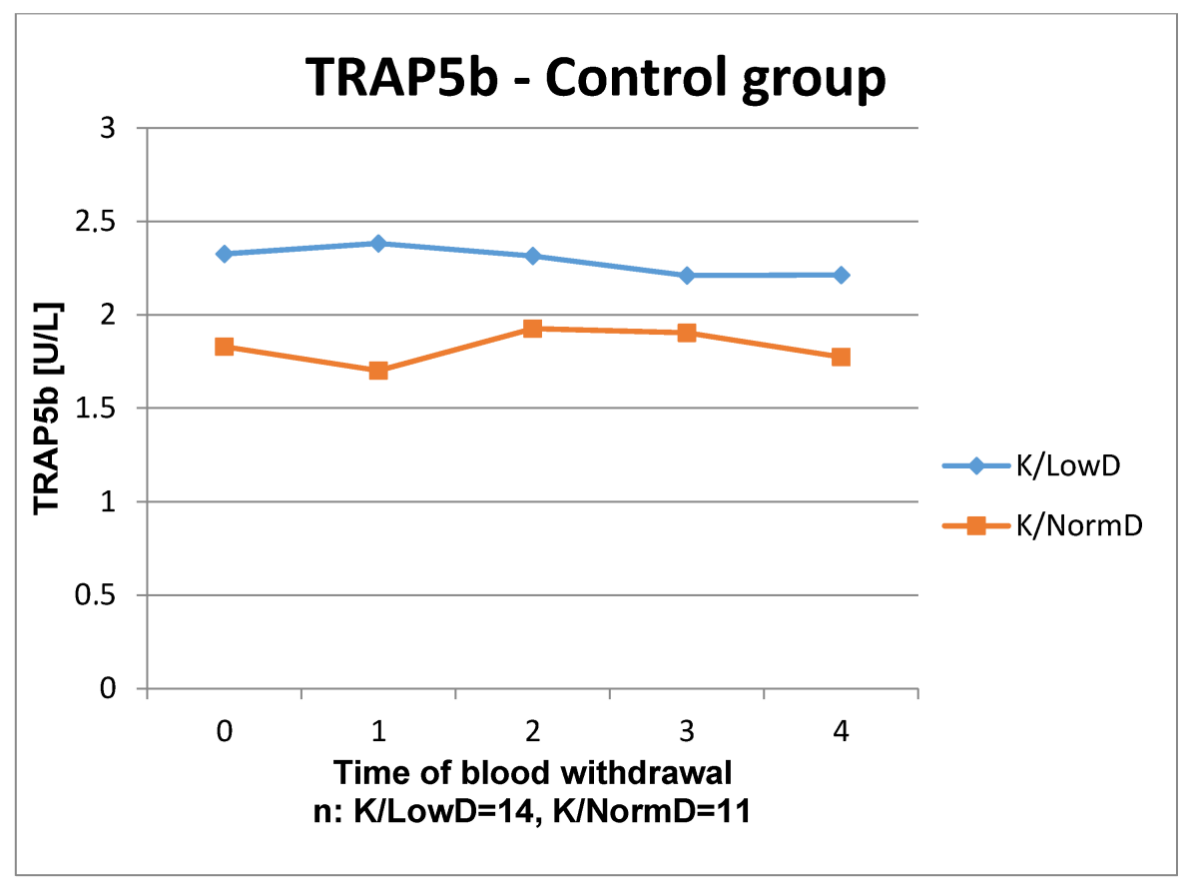
Time course of TRAP5b in patients without ESWT

**Figure 8:**
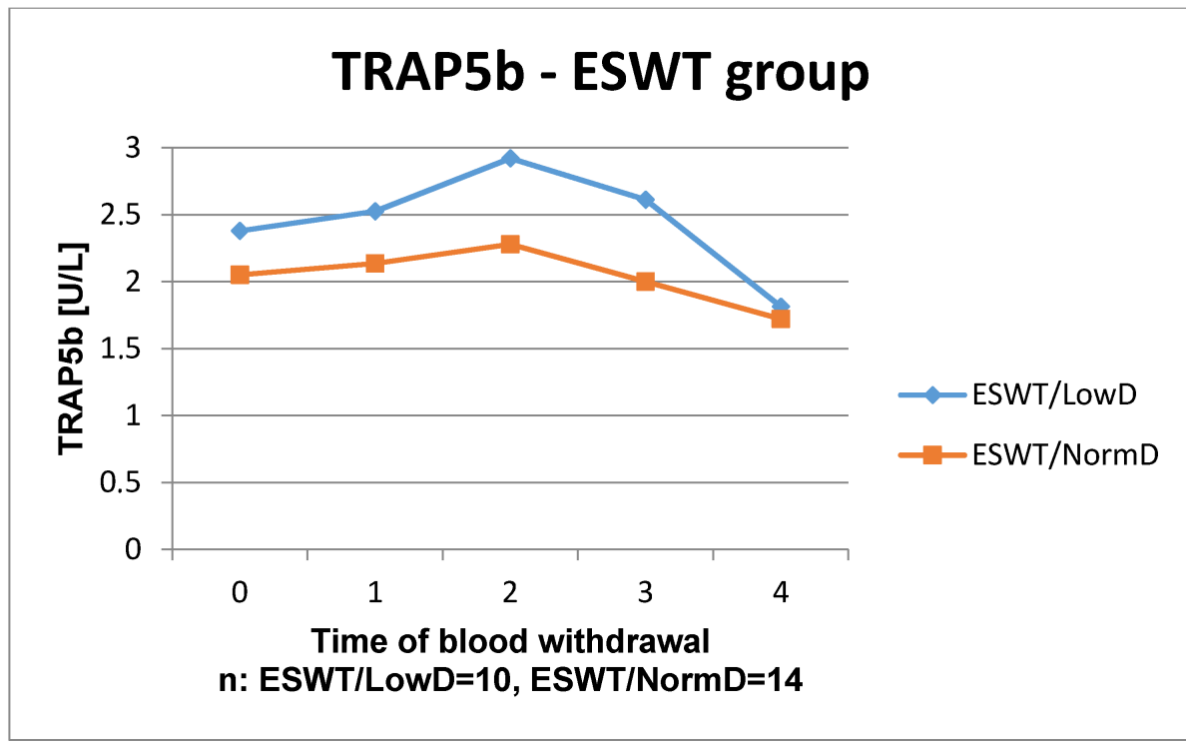
Time course of TRAP5b in patients with ESWT Reference range: F1.2–4.1 U/L, M 1.5–4.8 U/L

**Figure 9:**
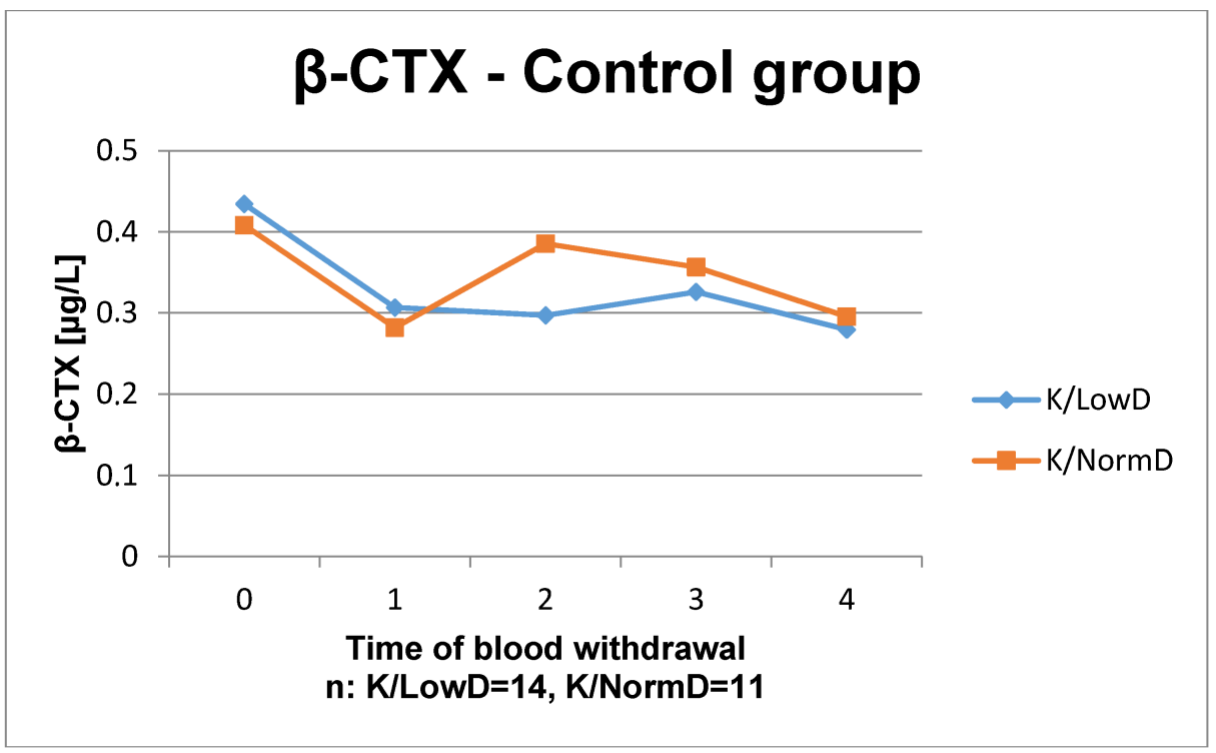
Time course of β-CTX in patients without ESWT

**Figure 10:**
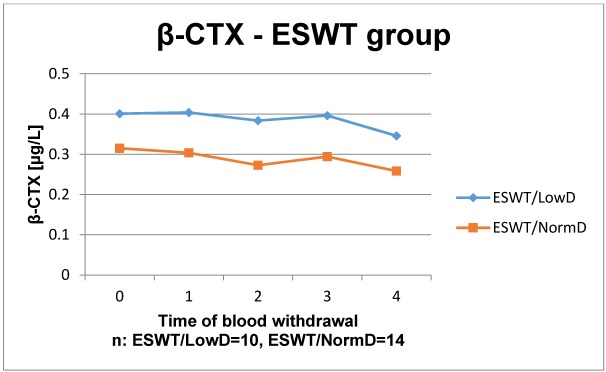
Time course of β-CTX in patients with ESWT Reference range: F: <0.57 µg/l, M: <0.58 µg/l–0.84 µg/l
